# Infection Related Glomerulonephritis Associated with *Staphylococcus epidermidis* in the Absence of Prosthetic Material

**DOI:** 10.1155/2014/130624

**Published:** 2014-08-04

**Authors:** Samer Mohandes, Eshetu Obole, Anjali Satoskar, Hari Polenakovik

**Affiliations:** ^1^Internal Medicine Department, Wright State University Boonshoft School of Medicine, Dayton, OH 45435, USA; ^2^Pathology Department, Ohio State University, Columbus, OH 43210, USA

## Abstract

We report a case of a 72-year-old diabetic male who developed infection-related glomerulonephritis (IRGN) in the setting of severe *Staphylococcus epidermidis* infection. He required renal replacement therapy for 6 weeks, but had full recovery of his kidney function with aggressive treatment of the infection. While this pathogen has been previously implicated as the cause of shunt nephritis, it is exceptionally rare to be associated with IRGN in the absence of a shunt or other prosthetic material.

## 1. Background 

Infection related glomerulonephritis (IRGN) is an immunologically mediated glomerular injury triggered by extrarenal infection. In the past, the majority of IRGN cases were related to infection with nephritogenic streptococcal strains [1, 2]. As the prevalence of* Streptococcus pyogenes *disease in developed countries has declined, other causative pathogens are recognized more frequently. A recent review of 86 patients with renal biopsy-confirmed IRGN identified* Streptococcus* spp. in 27.9% and* Staphylococcus* spp. in 24.4% of cases [1]. Adults over 55 years accounted for 54.6% of the cases, in contrast to previous studies (1974 and before), which reported IRGN to be a disease predominantly of children [3]. Emerging risk factors for IRGN identified in the recent studies include diabetes mellitus, alcoholism, HIV infection, malignancy, and injection drug use [1, 2, 4]. Whereas poststreptococcal GN typically occurs 1–4 weeks following a cutaneous infection, the majority of nonstreptococcal IRGN cases occur concurrently and at times precede the diagnosis of the infection. Therefore, the proposed term “IRGN” more accurately describes this pathology compared to the old term of “postinfectious GN” [4].* Staphylococcus epidermidis *is a well-established cause of shunt nephritis, accounting for 75% of cases [5]. In addition, IRGN cases associated with central venous catheter infections have been reported [6]. Herein, we describe a case of IRGN in the setting of severe spine infection with* S. epidermidis*, which occurred in the absence of foreign body.

## 2. Case Report 

A 72-year-old male with a history of type 2 diabetes mellitus and hypertension presented to the hospital with a 2-week history of progressive lower back pain and generalized weakness which culminated in inability to arise after a mechanical fall. On presentation he had a temperature of 36.7°C, BP 153/73 mm Hg, pulse 73 bpm, and respiratory rate 24/min. Pertinent physical exam findings included tenderness to palpation of the spine and left upper arm edema and tenderness. He recalled a difficult peripheral venipuncture from that arm 3 weeks prior to admission. Initial laboratory results were notable for leukocytosis (WBC 16,900/mm^3^ with 92.6% neutrophils), acute kidney injury (creatinine 1.6 mg/dL [144 *μ*mol/L] baseline 0.9 mg/dL [80 *μ*mol/L] and, BUN 57 mg/dL), hyponatremia (sodium 128 mmol/L), rhabdomyolysis (CK 3760 U/L), and abnormal urinalysis (specific gravity 1.025, large blood with sediment showing 6–10 RBC/hpf, WBC 11–20/hpf, moderate bacteria, and protein 100 mg/dL). Ciprofloxacin was initially started for a presumed urinary tract infection. Ultrasound of the left arm disclosed a 7 cm subcutaneous fluid collection, which was drained. Admission blood, urine, and left arm aspirate cultures grew methicillin-sensitive* S. epidermidis*. Magnetic resonance imaging of the spine revealed large subdural empyema and phlegmon extending from mid-thoracic to lumbar spine, nerve root inflammation, marked canal stenosis, and cord compression. He underwent emergent surgery for debridement of the infectious collections and T4-T5, T8-T9, and L4-L5 laminectomies. Transesophageal echocardiogram was negative for endocarditis. His postoperative course was complicated by acute blood loss anemia and hypotension attributed to hemorrhagic and septic shock requiring vasopressors and blood transfusion. He developed oligoanuria (responding to diuretics) and progressive increase in serum creatinine up to 5.7 mg/dL (504 *μ*mol/L), presumptively from acute tubular necrosis (ATN), necessitating initiation of renal replacement therapy. Due to persistent renal failure, a renal biopsy was performed.

**Figure 1:**
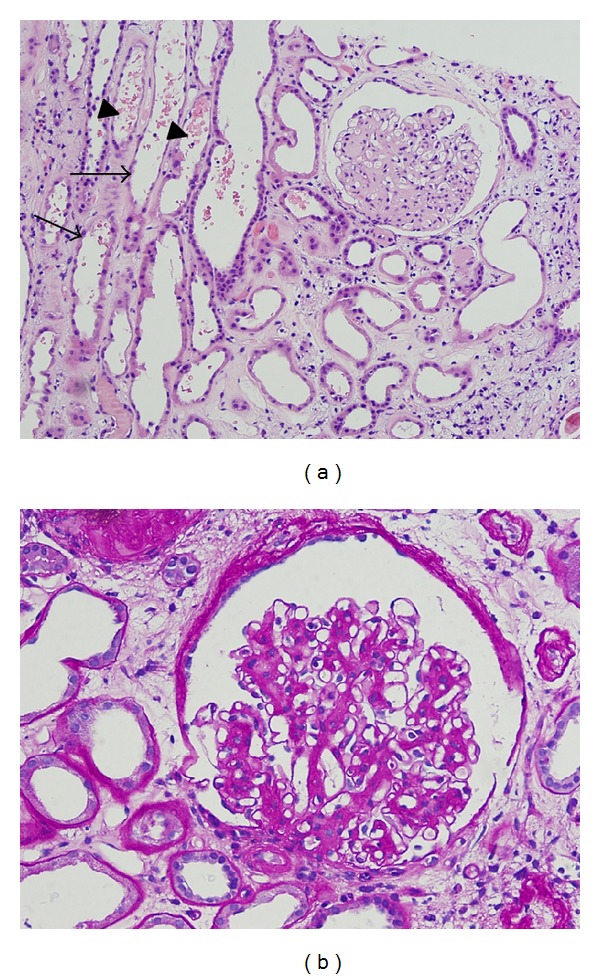
Light microscopy. (a) H&E staining demonstrating flat epithelium lining the tubules (arrows) consistent with ATN. RBCs in the tubules (arrowheads) suggest glomerular injury; (b) nodular mesangial expansion seen on PAS staining.

**Figure 2:**
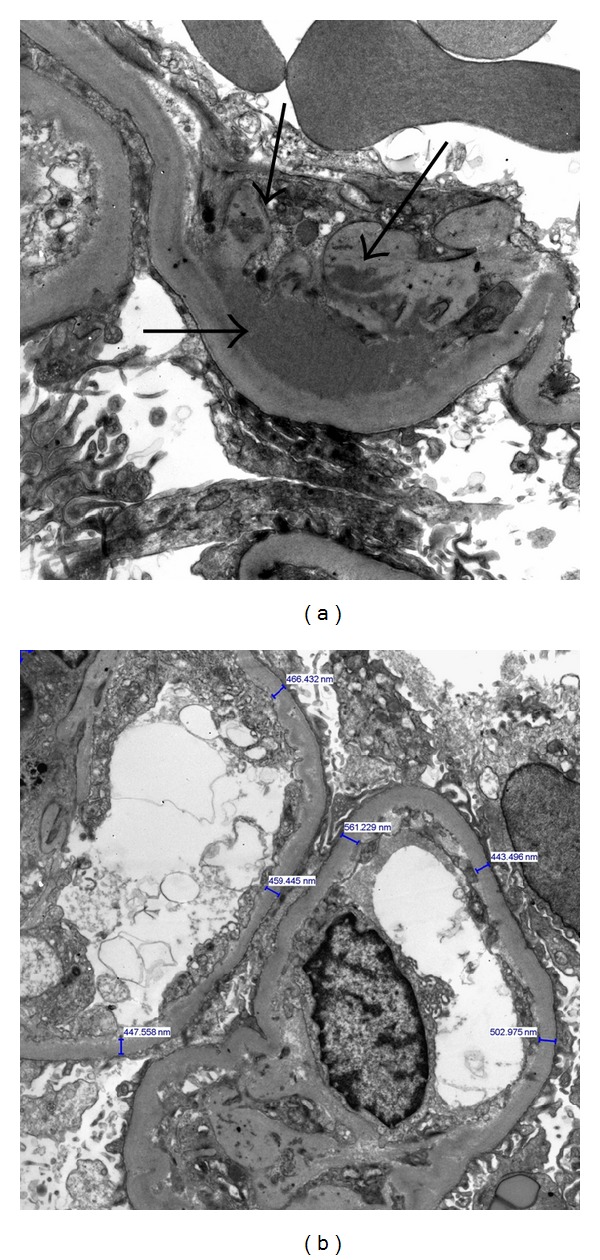
Electron microscopy. (a) Subepithelial glomerular deposits (arrows) suggesting an immunologic component to the glomerular injury; (b) thickening of the glomerular basement membrane consistent with an underlying diabetic glomerulopathy.

**Figure 3:**
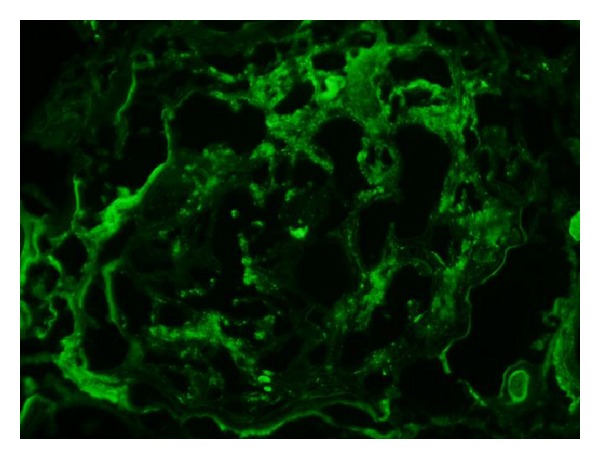
Immunofluorescence demonstrating granular deposits with C3.

Light microscopy (LM) (Figure 1) of the kidney specimen showed flattening of the tubular epithelium (Figure 1(a)) with tubular necrosis and edema suggesting acute tubular necrosis (ATN). Additionally, diffuse and nodular mesangial expansion (Figure 1(b)) as well as arteriolar hyaline were present consistent with an underlying diabetic nephropathy (DN). Mild patchy interstitial inflammatory cell infiltrates, predominantly mononuclear cells, were seen suggesting an underlying inflammatory process. Electron microscopy (EM) (Figure 2) showed mesangial and scattered subepithelial humps with electron dense immune type deposits (Figure 2(a)), moderate podocyte effacement, and thickening of the glomerular basement membrane at 506 ± 95 nm (normal 371 ± 56 nm) (Figure 2(b)). Direct immunofluorescence (IF) (Figure 3) showed mild mesangial staining of C3, IgA, and IgM. These findings were attributed to ATN and IRGN superimposed on DN.

There was gradual improvement of his renal function and after 6 weeks he was no longer hemodialysis (HD) dependent.* S. epidermidis *infection was aggressively treated with 8 weeks of ceftriaxone followed by 12 months of oral minocycline. At 12-month follow-up, his creatinine is 0.9 mg/dL (80 *μ*mol/L).

## 3. Discussion

*Staphylococcus epidermidis*, typically considered a low-virulence microbe, is a well-recognized cause of IRGN in association with prosthetic material, such as central venous catheters [6] and shunts [5]. However, severe* S. epidermidis *infection in the absence of prosthetic material is distinctly unusual. We hypothesize that our patient acquired the infection following a difficult peripheral venipuncture, which resulted in an infected hematoma. Immunosuppression, due to advanced age and underlying diabetes mellitus, facilitated hematogenous dissemination of* S. epidermidis *to the spine resulting in multifocal vertebral osteomyelitis as well as evolution of IRGN in the absence of prosthetic material. In addition to DN, superimposed IRGN delayed recovery from ATN and prolonged his dependence on HD.

On LM, IRGN manifests as mesangial expansion with hypercellularity. Electron dense immune deposits on EM are the hallmark of this disease. The distribution of these deposits varies depending on the stage of the disease but most commonly are found in the mesangium [1, 7]. Subepithelial deposits, classically seen in* S. pyogenes* IRGN, are also identified in* S. aureus* IRGN, however not as frequent in cases with comorbid DN [1]. On IF, predominant C3 and IgG deposits with minimal IgA, C1q, and IgM are seen [8]. Predominant IgA deposition mimicking IgA nephropathy is an emerging entity described with* S. aureus* and must be differentiated from primary IgA nephropathy [9, 10]. Codominant IgA deposition is commonly seen in IRGN [1] but is usually of mild to moderate intensity [7]. The kidney biopsy of our patient disclosed mesangial hypercellularity with electron dense immune deposits consistent with a mesangial pattern, likely representing a late stage of disease when the biopsy was performed. Only trace IgA was identified that was codominant with C3 and IgM, a finding consistent with reported cases of* S. aureus* associated IRGN [8]. ATN was the major finding on biopsy and appears to be the predominant cause of acute renal failure in this patient.

The mainstay therapy for IRGN is aggressive management of the infection and complications of nephritis [8]. Steroids and/or other immune-suppressives are generally not effective [8]. Only a minority of adults with IRGN will have full recovery of renal function. Our patient's good outcome was likely result of the aggressive surgical and medical treatment of his infection despite significant risk for poor outcome.

## 4. Conclusion

This case highlights the change in epidemiology of IRGN. Clinicians should consider IRGN when renal injury occurs in the course of* S. epidermidis *infection even in the absence of prosthetic material, especially when risk factors such as advanced age and diabetes mellitus type 2 are present. Aggressive treatment of infection is warranted and may lead to full recovery of renal function.
